# Stalk-derived carbon dots as nanosensors for Fe^3+^ ions detection and biological cell imaging

**DOI:** 10.3389/fbioe.2023.1187632

**Published:** 2023-04-28

**Authors:** Yongchao Du, Yaxi Li, Yunliang Liu, Naiyun Liu, Yuanyuan Cheng, Qiuzhong Shi, Xiang Liu, Zhimin Tao, Yumeng Guo, Jianguo Zhang, Najmeh Askaria, Haitao Li

**Affiliations:** ^1^ Institute for Energy Research, School of Chemistry and Chemical Engineering, Jiangsu University, Zhenjiang, China; ^2^ Institute of Medicine and Chemical Engineering, Zhenjiang College, Zhenjiang, China; ^3^ Jiangsu Province Key Laboratory of Medical Science and Laboratory Medicine, School of Medicine, Jiangsu University, Zhenjiang, China; ^4^ Zhenjiang Municipal Key Laboratory of High Technology for Basic and Translational Research on Exosomes, Zhenjiang, China; ^5^ Department of Critical Care Medicine, The Affiliated Hospital of Jiangsu University, Zhenjiang, China; ^6^ Guangxi Key Laboratory of Electrochemical Energy Materials, Guangxi University, Nanning, China

**Keywords:** carbon dots, detection, cell imaging, nanosensors, biomass

## Abstract

**Introduction:** Iron is one of the most important needed elements for the growth and reproduction of living organisms. The detection of iron levels is important and developing fluorescent probes with excellent sensitivity for Fe^3+^ ions is of great significance. Carbon dot (CDs) is a new type of fluorescent nanomaterial based on abundant and low-cost carbon elements. The use of widely distributed renewable agricultural waste straw as a carbon precursor to prepare CDs sensor can not only reduce the pollution caused by burning straw to the atmospheric environment, but also achieve the transformation of resources from waste to treasure.

**Methods:** In this study, CDs were obtained from corn stalk powder by pyrolysis and microwave process. The sensitivity and linear response range of CDs sensor was studied through analyzing the effect of different Fe^3+^ ions concentrations on the fluorescence quenching. The application of CDs in biological cell imaging was investigated using HGC-27 cells.

**Results:** The fluorescence quenching showed a good linear relationship with the Fe3+ concentration in the range from 0 to 128 μM, and a low detection limit of 63 nM. In addition, the CDs have high recognition for Fe3+ ions. Meanwhile, the CDs have a low cytotoxicity and desirable biocompatibility, allowing the multicolor living cell imaging.

**Conclusion:** The prepared CDs can be used as fluorescent sensors for the selective detection of Fe^3+^ ions and biological cell imaging. Our results supported that the conversion of agricultural waste into carbon nanomaterials has great potential to be developed.

## 1 Introduction

Iron is one of the most important needed elements for the growth and reproduction of living organisms. Iron is widely distributed on the earth, playing an important role in oxygen transport (Dutt et al., 2022), enzymatic reactions (Stoyanovsky et al., 2019), cellular metabolism (Soares and Hamza, 2016), electron transport (Gupta et al., 2021) and DNA synthesis (Li et al., 2021). Although iron plays a key role in these aspects, too much iron will be toxic to our bodies due to the generation of free radicals, which results in the destruction of biological macromolecules (Wang and Babitt, 2019). Moreover, excessive levels of iron can cause imbalances in the intracellular environment as well (Navazesh and Ji, 2022). The dual function of iron highlights the importance of maintaining a strict balance of iron levels in the body (Duck and Connor, 2016). Therefore, the detection of iron levels is greatly important. Currently, UV spectrophotometry (Cheng et al., 2022a), atomic absorption (Zvěřina et al., 2019) and inductively coupled plasma mass spectrometry (ICP-MS) (Wheal et al., 2016) are mainly used for the detection of iron ions. However, these methods have numerous disadvantages, such as tedious, time-consuming operation, low recovery, small detection range of solution pH, easy to be interfered by other metal ions and high testing costs (Wu et al., 2019). Thus, a number of methods based on fluorescence analysis have been developed to detect elemental iron (Lin et al., 2017; Duan et al., 2022). Fluorescence spectrophotometry is widely used for the analysis and detection of substances because of its simplicity of operation, high sensitivity and low cost (Yang et al., 2015; Mehta et al., 2021; Liu et al., 2023).

Carbon dot (CDs) as a new type of fluorescent nanomaterial based on abundant and low-cost carbon elements have achieved great attention (Liu et al., 2016; Chen et al., 2020; Yan et al., 2022). Compared with traditional semiconductor quantum dots and organic fluorescent dye molecules, CDs have the following advantages: high fluorescence intensity, high quantum yield, excellent selectivity, suitable biocompatibility, low toxicity, etc. (Park et al., 2020; Nagaraj et al., 2022; Wang and Lu, 2022) Therefore, CDs are widely used in analytical detection, biosensing and other fields (Keerthana et al., 2022). There are two current methods for CDs synthesis, including top-down and bottom-up processes. The top-down method is to “fracture” carbon material precursors (such as graphite), usually under harsh conditions, and requires a long synthesis time (Li et al., 2015; Perumal et al., 2021). In the bottom-up process, a large number of molecular precursors can be used as carbon sources, and uniform CDs can be obtained through microwave and hydrothermal process, etc. (Yang et al., 2013; Singh et al., 2019; Rani et al., 2020) Among them, the microwave synthesis method has gained increasing attention because of its fast reaction rates, simplicity, green and low energy consumption (Rodríguez-Padrón et al., 2018; Karakoçak et al., 2021). In addition, the molecular precursors of CDs are usually citric acid (Liu et al., 2017), glucose (Ferreyra et al., 2020), and various amino acids (Kang and Kang, 2021). However, the process of the aforementioned synthesis methods is tedious, high cost with long dialysis time.

In recent years, a large number of studies have been carried out using biomass as natural precursors for the synthesis of CDs (Wareing et al., 2021). Crop straw is renewable, in line with the concept of green chemistry and sustainable development, and is a green and friendly carbon material. After crop harvest, straw is usually left idle and wasted or burned on site, which will not only cause air, soil and water pollution, but also waste the significant resources (Zhang et al., 2011). In fact, a large portion of crop nutrients are stored in the straw, being rich in organic matter and nutrients such as nitrogen, phosphorus and potassium. Meanwhile, crop straw is rich in lignin and cellulose, and the rich unsaturated bonds are more conducive to the formation of graphite carbon lattice in CDs (Mittal et al., 2017). Therefore, straw is a valuable biological resource with reuse value.

This research presents a method to prepare CDs nanosensors by microwave using crop straw product (CSP) as the carbon source. The synthesized CDs exhibited a large Stokes shift (about 100 nm) when excited by UV light at 365 nm. The surface of CDs was rich in -COOH and -NH_2_, forming stable chelates with Fe ligands and thus causing fluorescence quenching of CDs. This quenching effect is influenced by the Fe^3+^ ions concentration, which provides a basis for studying the detection of Fe^3+^ ions concentration in the environment. This study also investigated the application of CDs in biological cell imaging using HGC-27 cells. Based on the results, the fluorescent CDs have desired biocompatibility and low toxicity, which will lay the foundation for their application in bioanalytical and biomedical science.

## 2 Experimental section

### 2.1 Chemicals and materials

CSP was obtained from a local supplier and used as required. Sodium sulfide, methanol, and hydrogen peroxide were purchased from Sinopharm Chemical Regent Co., Ltd. All chemical reagents were used without further processing and purification. The water used in this experiment was ultrapure water.

### 2.2 Synthesis of CDs and characterization

**FIGURE 1:**
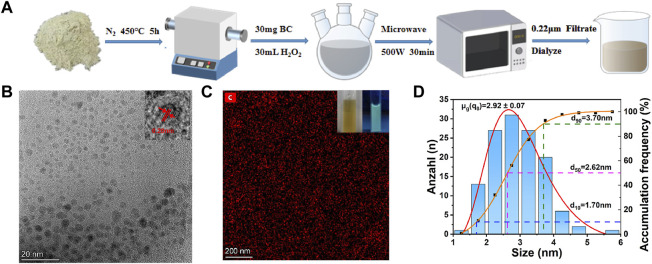
**(A)** The synthetic scheme of CDs from CSP. **(B)** TEM images of CDs. Inset is the high-resolution TEM image. **(C)** EDX mapping images of CDs. Insets are the photographs of the obtained CDs solution illuminated under sunlight and a UV lamp (365 nm), respectively. **(D)** Particle size distribution of CDs.

CDs were produced from CSP by pyrolysis and microwave methods. The synthesis steps are shown in Figure 1A. For the easier generation of CDs, the CSP was simply pretreated, resulting in its internal structure disruption. Then, the pretreated CSP was transferred to a tube furnace and calcined at 450°C for 5 h under a nitrogen atmosphere to obtain black carbon powder (BCP) as the precursor of CDs. Thereafter, 30 mg of BCP was added to 30 mL of 30% H_2_O_2_ and the mixture was reacted in a microwave reactor for 30 min (500 W). Finally, the dark brown CDs solution was collected from the supernatant and stored at 4°C for further use. The condensed solution was filtered (0.22 μm), dialysis (100Da) and further characterized.

Transmission electron microscopy (TEM) images were obtained on a HITACHI H-8100 electron microscopy (Hitachi, Tokyo, Japan) operated at 200 kV. The X-ray diffraction (XRD) patterns were investigated by a LabX XRD-6100 X-ray diffractometer with Cu Kα radiation (40 kV, 30 mA) of wavelength 0.154 nm (SHIMADZU, Japan). The absorbance data of spectrophotometer were measured by SHIMADZU UV-2,700 ultraviolet-visible (UV-Vis) spectrophotometer. Fourier-transform infrared spectra (FTIR) were measured by Nicolet iS50 FTIR (Thermo Fisher Scientific, United States). Raman spectroscopy was conducted using a confocal Raman system (RTS2, Zolix) with a 532 nm excitation source. The PL spectra were recorded at an excitation wavelength of 365 nm at room temperature using a photoluminescence spectrometer (Perkin Elmer, United States).

### 2.3 Photoluminescence spectra

The test of Fe^3+^ ions detection was investigated at room temperature by adding the Fe^3+^ ions related salt to the CDs solution (0.05 mg/mL). First, 0.337 g FeCl_3_·6H_2_O was dissolved in 10 mL ultrapure water to obtain a 5.12 mM Fe^3+^ ions solution. Then, the solution was diluted with ultrapure water to obtain a series of Fe^3+^ ions solution with the concentration of 2.56 mM, 1.28 mM, 640 μM, 320 μM, 160 μM, 80 μM, 40 μM, 20 μM, 10 μM, 5 μM, 1 μM and 500 nM, respectively. These Fe^3+^ ions solution was added in sequence with a specific amount to the previous CDs solution to obtain the CDs solution with a series of Fe^3+^ concentrations of 1 μM, 2 μM, 4 μM, 8 μM, 16 μM, 32 μM, 64 μM, 128 μM. For example, for obtaining the 10 μM Fe^3+^ ions in CDs, 200 μL of 100 μM Fe^3+^ ions solution was diluted with 2 mL of previous CDs. Then the solution was investigated in fluorescence spectrometer for the emission spectra from 380 nm to 600 nm, with the excitation wavelength of 365 nm and the slit width of 10 nm.

The parameters of the photoluminescence spectrometer were set as follows: Chopper Speed (Hz) = 10, Excitation Wavelength (nm) = 365, Excitation Slit (nm) = 10, Excitation Filter = Air, Emission Slit (nm) = 10, Emission Filter = Air, PMT Voltage(V) = 550, PMT Gain = x1, Response Width (nm) = 10.

### 2.4 Quantum yield measurements

The quantum yield (QY) of the CDs was calculated by the following equation:

$$\Psi_{x}=\Psi_{s}\frac{I_{x}}{I_{s}}\frac{A_{s}}{A_{x}}\frac{\eta_{x}^{2}}{\eta_{s}^{2}}$$

Where, $\Psi_{x}$ is the fluorescence quantum yield; x and S represent the test substance and reference compound. Quinine sulfate dissolved in 0.05 M H_2_SO_4_ (*ψ*_s_ = 0.54) was chosen as the reference. *η* is the refractive index (1.33 for aqueous solution). A is the absorbance at the excitation wavelength of 365 nm and I is the integrated fluorescence intensity in the fluorescence emission spectrum.

### 2.5 Cytotoxicity assay

The cytotoxicity of CDs was determined using the CCK8 method. HGC-27 cells were inoculated in 96-well plates with a final volume of 100 μL. Each group is set with 8 cell number gradients, and each gradient is set with three replicate wells, a total of 2 groups (sample group to be assayed and qualified sample group as control). The cell number gradients in each group were: 0, 400, 800, 1,600, 3,200, 6,400, 12,800 and 25,600 cells/well, respectively. 10 μL CCK-8 solution was added to each well at the end of plate laying and incubated at 37°C for 2 h. Then, 0.05 mg/mL of CDs solution was added to each group of samples and incubated for 24 h and 48 h, respectively. The absorbance of the synthesized well contents was monitored at 450 nm using an enzyme marker (Permanent Medical, DNM-9606). Relative cell viability was determined by comparison with the control. These experiments were repeated three times. The results are presented as mean ± standard deviation. The cell viability was calculated by following equation (Fu et al., 2021):

$$Cell\;viability\;\left(\%\right)=\left(the\;absorbance\;of\;the\;experimental\;group/the\;absorbance\;of\;the\;control\;group\right)\times 100\%$$

### 2.6 Cell imaging

The combined effect of CDs in HGC-27 cells was investigated. HGC-27 cells were cultured in DMEM culture medium with 10% (v/v) fetal bovine serum and 1% penicillin-streptomycin. Approximately 2 × 105 HGC-27 cells were seeded in culture dishes (diameter: 40 mm) and incubated for 24 h at 37°C under an atmosphere of 5% CO_2_. The synthetic CDs (0.05 mg/mL) were then mixed with cell cultures and further incubated at 37°C for 1 h. The cells were washed with PBS three times and examined under a confocal microscope (Zeiss, LSM 880 NLO) using the 405 nm laser.

## 3 Results and discussion

### 3.1 Characterization of CDs

The CDs was prepared from CSP by pyrolysis and microwave processes (Figure 1A). The TEM images (Figure 1B) were used to study the morphology of the CDs, and the inset shows that the lattice spacing of CDs is about 0.20 nm. As shown in Figure 1C, the uniform distribution of C element in CDs is determined, and the existence of its related elements is proved. As can be seen from the inset of Figure 1C, the CDs solution is transparent and homogeneous yellowish solution under visible light, while it emits blue-green fluorescence under UV light at 365 nm. The CDs have uniform dimensions in the size range of 1.5–6 nm as illustrated in Figure 1D, and the average particle size of CDs is 2.92 nm. 90% of CDs are below 3 nm, 50% of CDs are below 2 nm, and only 10% of CDs are below 1 nm.

**FIGURE 2:**
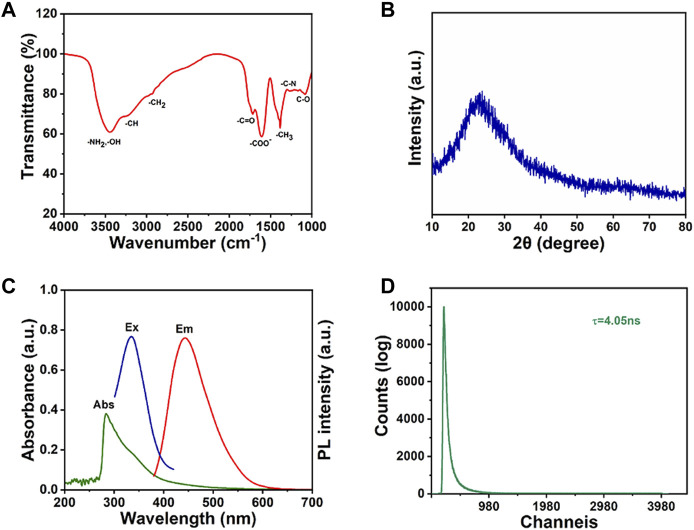
**(A)** FTIR spectrum, **(B)** XRD spectrum, **(C)** UV absorption, excitation and emission spectra and **(D)** fluorescence lifetime spectrum of CDs.

The CDs surface functional groups were further determined by FTIR spectrum (Figure 2A). The peak at ∼1,072 cm^−1^ is attributed to the C-O stretching vibration and the peak at ∼1,265 cm^−1^ is contributed to the torsional vibration of the -CH_2_ or the stretching vibration of the C-N (Long et al., 2022).^40^ The strong peak at ∼1,384 cm^−1^ is contributed to the symmetric variable angle of the -CH_3_. The peak at 1,616 cm^−1^ is due to the antisymmetric stretching of -COO^-^. The peaks at 1732, 2,927 and 3,220 cm^−1^ are attributed to the stretching vibration of C=O, the antisymmetric stretching of -CH_2_ and the stretching vibration of -CH, respectively. The strong peak at 3,300 cm^−1^ is attributed to the O-H stretching vibration and the anti-symmetric stretching of -NH_2_ (Cheng et al., 2022b). This indicates the presence of a large number of carboxyl groups and other oxygen-containing functional groups on the surface of CDs, resulting in beneficial water dispersion of CDs (Naciri et al., 2022). Oxidation causes the introduction of C=O-containing groups, such as -COOH, on the surface of CDs. The Raman spectrum of CDs exhibits two distinct carbon peaks (Supplementary Figure S1) (Li et al., 2019). In addition, XRD analysis reveals a broad diffraction peak at 22.5°, indicating that the CDs structure is disordered amorphous, as shown in Figure 2B (Xu et al., 2021).

The optical properties of as-prepared CDs were then investigated. The UV absorption, excitation and emission spectra of CDs are shown in Figure 2C. The CDs solution typically shows strong absorption in the UV region with the maximum absorption peak at 284 nm, while the tail extends into the visible absorption range. The peak of 284 nm is attributed to π→π* electron transitions (Ezati and Rhim, 2022). The CDs shows the maximum excitation and the emission wavelengths at 326 nm and 445 nm, respectively. In addition, the fluorescence lifetime of CDs can reach as long as 4.05 ns (Figure 2D).

**FIGURE 3:**
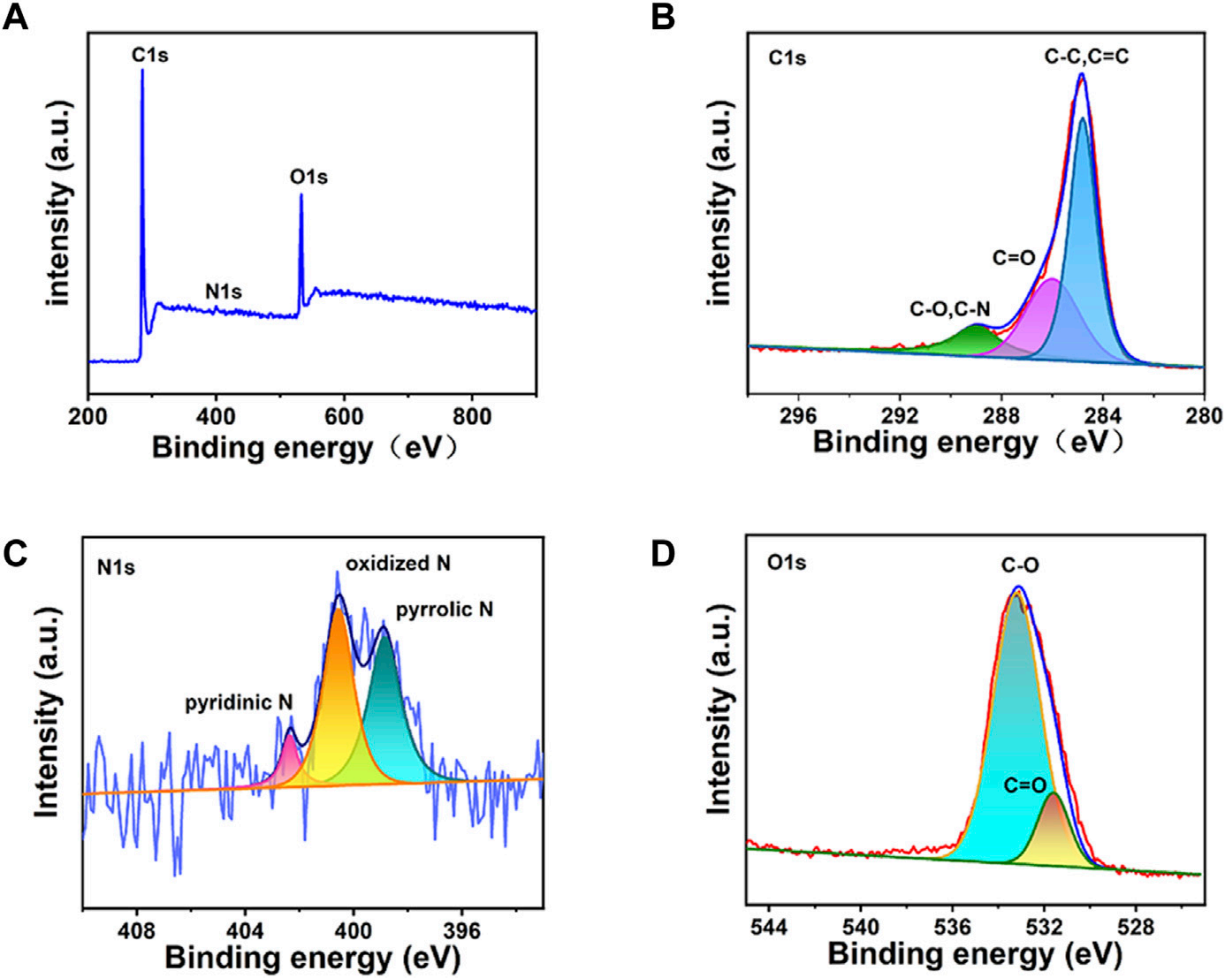
The XPS spectra of the CDs: **(A)** the total spectrum, **(B)** C1s enlarged region, **(C)** N1s enlarged region, **(D)** O1s enlarged region.

XPS characterization was used to further investigate the structure of CDs. In Figure 3A, three obvious peaks are observed regarding to C1s, N1s and O1s at the binding energy of 284.8, 400, and 533 eV, respectively. For the high-resolution XPS pattern of C1s (Figure 3B), three peaks at 284.9, 286.1, and 288.5 eV are attributed to [C-O, C-N], C=O and [C-C, C=C] groups, respectively (Zhao et al., 2020; Liu et al., 2021b). In the N1s XPS spectrum of CDs (Figure 3C), three peaks are observed with binding energies of 399, 400.5, and 402.5 eV, corresponding to pyridine nitrogen, nitrogen oxide and pyrrole nitrogen, respectively (Li et al., 2022a). The presence of the -NH_2_ structure was further confirmed. Based on the O1s XPS spectrum (Figure 3D), two main peaks located at 533.1 and 531.5 eV indicate the presence of C-O and C=O groups, which coincided with the C1s spectrum (Li et al., 2022b).

### 3.2 Fluorescence detection

**FIGURE 4:**
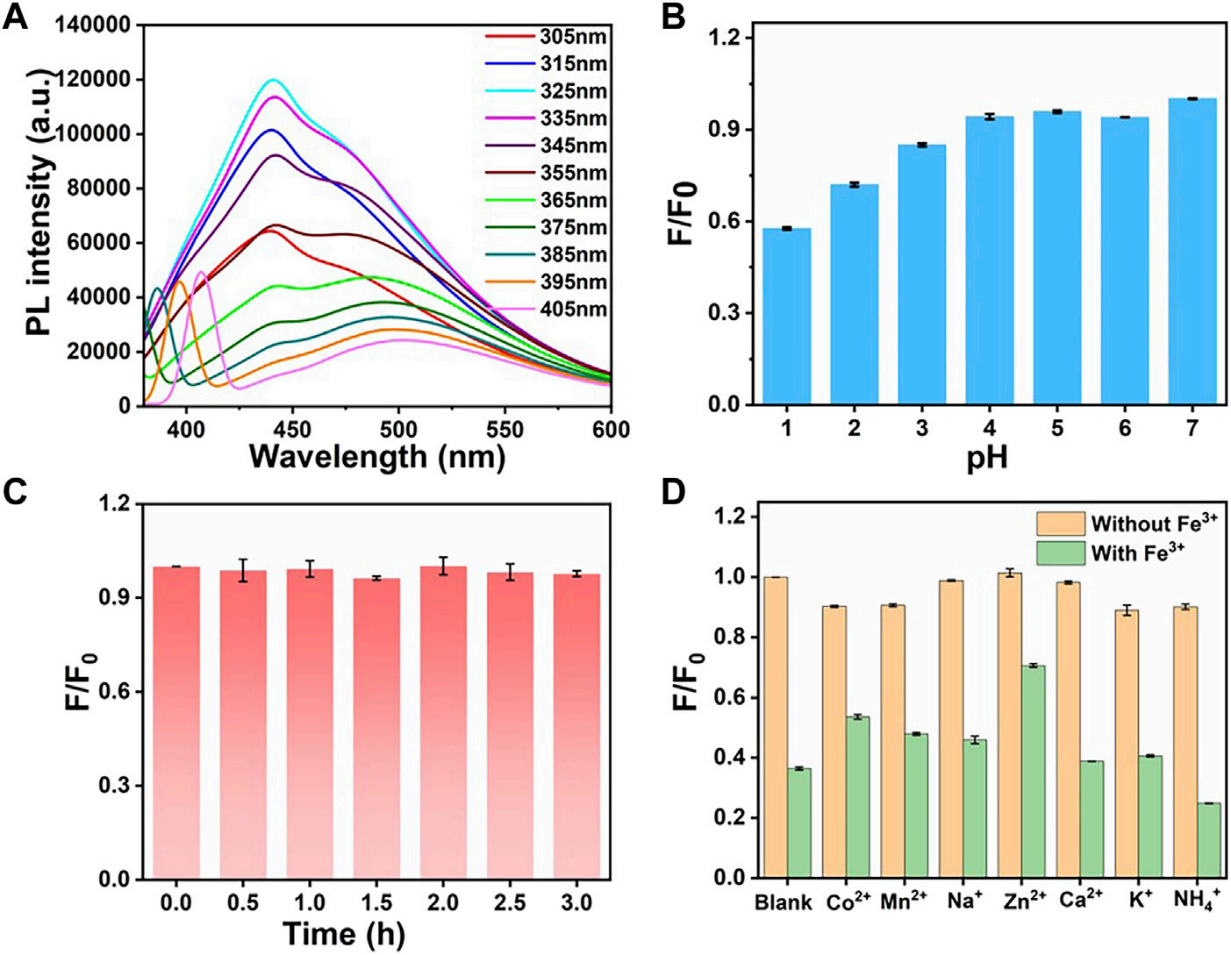
**(A)** Fluorescence emission spectra of CDs at different excitation wavelengths. **(B)** Fluorescence intensity of CDs under different pH conditions. **(C)** Fluorescence intensity changes of CDs after being irradiated by 300 W xenon lamp for different time. **(D)** Fluorescence intensity changes in the presence of different metal ions (yellow) and after the addition of Fe^3+^ ions (green). For panel B–D, F_0_ and F are the fluorescence intensities of the CDs before and after treatment, respectively.

In this work, we investigated the sensitivity and linear response range of this metal ion sensor by preparing a series of Fe^3+^ ions solutions with different concentrations and mixed with CDs. The fluorescent properties of CDs were studied in detail firstly. With the increase of excitation wavelength, the fluorescence intensity first increased and then decreased. The maximum value was reached at the excitation wavelength of 325 nm (Figure 4A). The position of the fluorescence emission peak gradually increased, which may be due to the non-uniform particle size of carbon particles in the CDs solution. At 365 nm excitation, the characteristic emission maximum at 480 nm appeared with a fluorescence quantum yield of 2.1% using rhodamine B as a reference.

The effects of the pH of the solution on the stability of fluorescence intensity were investigated as well (Figure 4B). Under acidic conditions, the fluorescence intensity decreased gradually with pH decreasing. It may be due to the combination of nitrogen-containing functional groups in the CDs solution with H^+^ at lower pH values. The introduction of H^+^ leads to the weakening of the fluorescence intensity of CDs. Considering that the pH of the physiological environment is 7–8, the fluorescence intensity in this range is relatively stable and favourable for biofield applications. As shown in Figure 4C, the stability of CDs at different time is obtained. The fluorescence intensity of CDs remained highly stable after being irradiated by 300 W xenon lamp for 3 h. The synthesized CDs shows long-term stability in ultrapure water for more than 2 weeks. As can be seen in Supplementary Figure S2, at room temperature, the fluorescence intensity of the CDs solution remained almost unchanged after 2 weeks, proving that it has good stability, and thus it can be used as a fluorescent probe under optimal conditions.

The effect of different metal ions on the fluorescence intensity of CDs solution was further studied. Different cations such as Na^+^, NH_4_^+^, K^+^, Ca^2+^, Mn^2+^, Fe^3+^, Co^2+^, Ni^2+^, Zn^2+^ ions were added to the CDs solution, respectively (Supplementary Figures S3–S7). After each cation solution was added to the CDs solution and mixed thoroughly for 10 min, separately, the fluorescence changes were recorded (red bar). As can be seen in Figure 4D, the other monovalent cations and divalent metal ions have little effect on the fluorescence intensity of CDs, while the addition of Fe^3+^ ions caused a significant quenching of the fluorescence (orange bar). It can be demonstrated that the CDs have high recognition for Fe^3+^ ions instead of other cations. The addition of Fe^3+^ ions immediately induced a fluorescence quench, which can be attributed to the paramagnetic nature of Fe^3+^ ions and can be used as a fluorescence quencher (Liu et al., 2021a).

**FIGURE 5:**
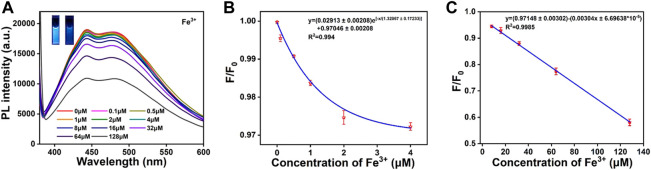
**(A)** Fluorescence emission response of CDs solution (0.05 mg/mL) with different concentrations of Fe^3+^ ions (0–128 μm). The inset shows the photographs of CDs solution (left) and CDs-Fe^3+^ complexes (right) under 365 nm UV irradiation. **(B)** When the Fe^3+^ ions concentration is 0–4 μM, linear correlation between F/F_0_ and Fe^3+^ ions concentration. **(C)** When the Fe^3+^ ions concentration is 4–128 μM, the linear correlation between F/F_0_ and Fe^3+^ ions concentration. F_0_ and F are the fluorescence intensity for CDs solution in the absence and presence of corresponding ions, respectively.

According to the results shown in Figure 5A, the fluorescence intensity of CDs was decreased with the Fe^3+^ ions concentration increasing. From the inset of Figure 5A, it can be seen that there is a significant decrease in the brightness of the CDs solution under the UV lamp irradiation at 365 nm. The fluorescence emission intensity decreases by 43% when the Fe^3+^ ions concentration increases from 0 to 128 μM. As shown in Figure 5B, the fluorescence intensity of CDs is strongly correlated with different concentrations of Fe^3+^ ions. This paves the way for qualitative and quantitative detection of Fe^3+^ ions concentrations in the real environment. According to the Stern–Volmer theoretical equation:

$$F_{0}/F=1+K_{SV}C_{q}$$

Where Ksv is the bursting constant, C_q_ is the concentration of the quencher (Fe^3+^), F_0_ is the maximum fluorescence emission intensity of CDs without Fe^3+^ ions addition, and F is the maximum fluorescence emission intensity of CDs with Fe^3+^ ions addition. When the concentration of added Fe^3+^ ions is 0–4 μM, the linear regression equation is as follows (Figure 5B):

$$F/F_{0}=\left(0.02913\pm 0.00208\right)e^{\left[-C_{{Fe}^{3+}}/\left(1.32067\pm 0.17233\right)\right]}+\left(0.97046\pm 0.00208\right),R^{2}=0.994$$

When the concentration of added Fe ions is 4–128 μM, the linear regression equation is as follows (Figure 5C):

$$F/F_{0}=\left(0.97148\pm 0.00302\right)-\left(0.00304C_{{Fe}^{3+}}\pm 6.69638\times 10^{-5}\right),R^{2}=0.9985$$

The regression coefficient of correlation was found to be 0.994 and 0.9985, respectively. Based on LOD = 3σ/S (σ is the standard deviation of 12 blanks, S is the slope of the obtained linear relationship), the limit of detection of Fe^3+^ ions concentration is calculated as 63 nM. The prepared CDs in this work, not only have the maximum absorption peak at 480 nm, but also have a large Stokes shift, better stability, higher selectivity and lower detection limits. These advantages suggest that they can be used as fluorescent probes for the detection of Fe^3+^ ions.

In order to investigate the practical feasibility of CDs, the concentration of Fe^3+^ in the actual water samples of tap water was tested. An absolute concentration of Fe^3+^ ions was added in the tap water, then the fluorescence intensity was measured and the Fe^3+^ ions concentration was calculated through the linear regression equation. The Fe^3+^ concentration in tap water was measured by fluorescence quenching method and inductively coupled plasma-mass spectrometry (ICP-MS) as well (Supplementary Figure S8). In this method, the concentration of added Fe^3+^ ions standard solutions were 8.00 μM and 16.00 μM and the measured values were 8.09 μM and 16.10 μM, respectively. The measured recovery rate of Fe^3+^ ions by this method was between 95%–98%, and the standard deviation was less than 1.7% (Table 1). The above results show that the detection results of this method have adequate accuracy and can be used to detect the content of Fe^3+^ ions in tap water.

**TABLE 1:** Results for the determination of Fe^3+^ of tap water.

Sample	Added Fe^3+^ (μM)	Detected Fe^3+^ (μM)	Recovery (%)	RSD (%), n = 3
Tap water	0	0.46	0	0
	8.00	8.09	95.3	1.7
	16.00	16.10	97.8	1.6

### 3.3 Fluorescence detection mechanism

**FIGURE 6:**
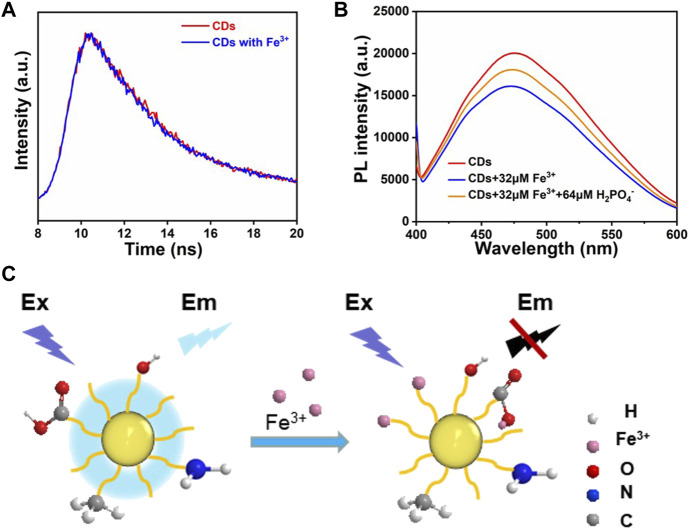
**(A)** The fluorescence decay curves of the CDs and CDs with Fe^3+^. **(B)** Comparison of fluorescence intensity after the addition of Fe^3+^ ions and H_2_PO_4_^−^ ions to CDs solution. **(C)** Fluorescence quenching mechanism of CDs.

The fluorescence quenching mechanism was investigated in some detail. To distinguish between the dynamic and static quenching modes clearly, fluorescence lifetime measurements for the CDs were conducted. The average fluorescence lifetime of CDs has no obvious change in the absence of Fe^3+^ (Figure 6A), which seems to indicate that the fluorescence decay is ascribed to the static quenching (Wahba et al., 2015; Zhang et al., 2022). Therefore, the high selectivity of CDs to Fe^3+^ may be attributed to the fact that compared with the other metal ions, the Fe^3+^ has the stronger binding affinity and faster chelating kinetics with carboxylic groups and N-containing function groups on the surfaces of the CDs (Li et al., 2017; Zhang et al., 2021). From the FTIR and XPS analysis, it can be found that the surface of the CDs was covered with a large number of functional groups (-NH_2_, -OH, -COOH). As an electron donor, the N atoms have a sufficient negative charge, easily losing its outermost electron, which promotes the complexation reaction between Fe^3+^ ions and the functional groups on the surface of CDs. In addition, the CDs solution was treated with an appropriate amount of H_2_PO_4_^−^ ions to further investigate the interaction between CDs and Fe^3+^ ions. The H_2_PO_4_^−^ introduced can compete with CDs and form complexes with Fe^3+^ ions. The results show that the fluorescence intensity of the Fe^3+^ ions/CDs solution can be restored to different degrees (Figure 6B). This may be due to the larger coordination constants of Fe^3+^ ions and H_2_PO_4_^−^ ions, which leads to the recovery of the fluorescence intensity of the CDs solution (Figure 6C). It is reasonable to assume that the occurrence of fluorescence quenching may be due to the formation of non-fluorescent complexes between the CDs surface functional groups and Fe^3+^ ions.

### 3.4 Sensor application

**FIGURE 7:**
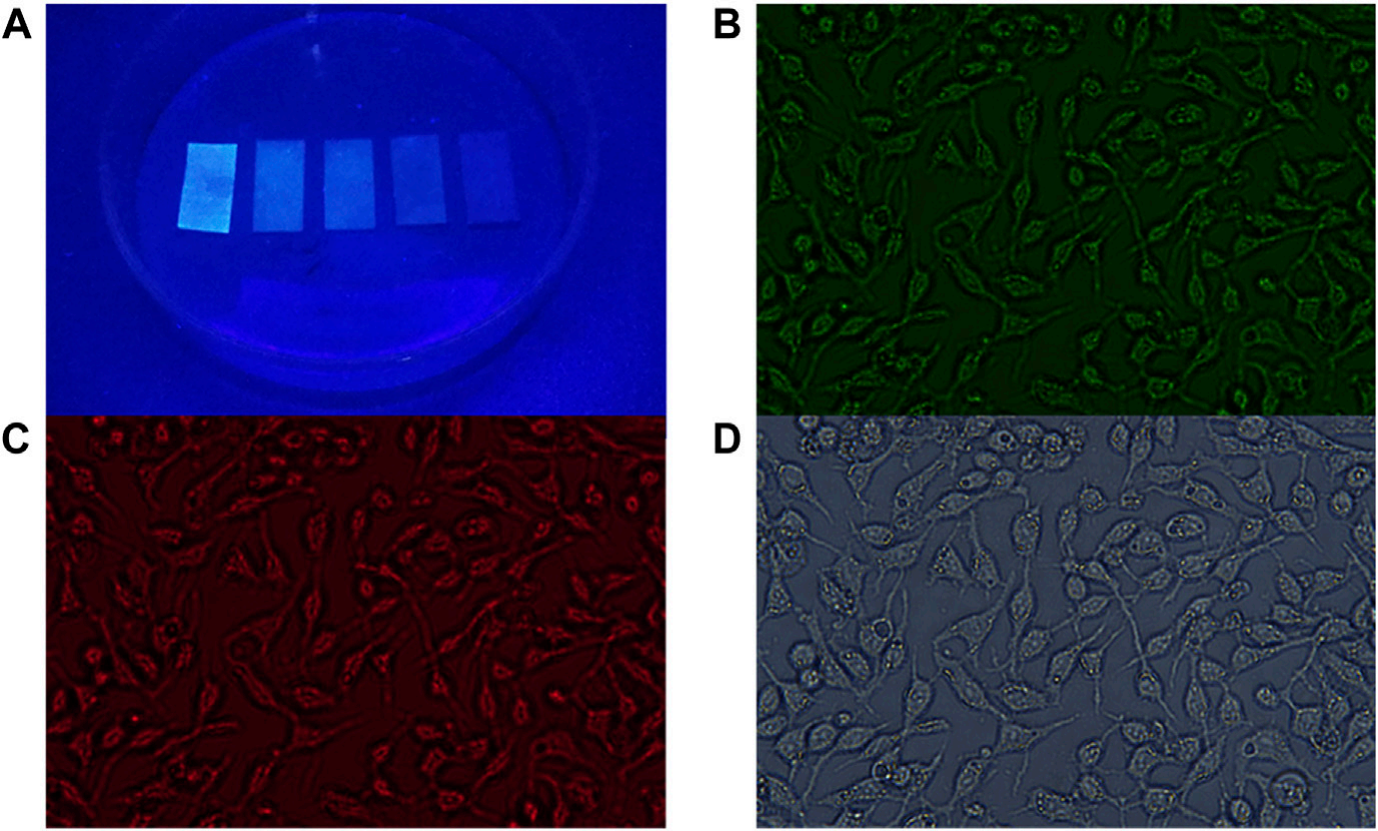
**(A)** Captured PL emission of CDs in series concentrations of Fe^3+^ ions solution (0, 10, 50, 100, and 500 μM from left to right). Confocal laser scanning microscopy images of CDs in cells **(B)** excitation wavelength of 465–495 nm; emission was collected at 512–552 nm; **(C)** excitation wavelength of 540–580 nm; emission was collected at 600–660 nm; **(D)** bright field.

The principle of PL emission was implemented in the application as Fe detection paper. First, the CDs solution was dropped on non-fluorescent glass fiber test paper. After natural drying, Fe^3+^ solutions of different concentrations were dropped into the test papers. Subsequently, the test papers were placed under a UV lamp and the corresponding emission light was captured at 480 nm. As shown in Figure 7A, different test papers exhibit different intensities of emitted light, which provides an effective and convenient method for qualitatively testing the content of Fe^3+^ ions in water. This method can be used for water quality testing in remote areas without advanced instruments. From the above results, the sensor demonstrated proper sensitivity to Fe^3+^ ions under fluorescence emission, and the color change was seen in Fe^3+^ ions solutions ranging from 0 μM to 500 μM (Figure 7A).

### 3.5 Cytotoxicity and living cell imaging

The prepared CDs present desirable optical properties, therefore the prepared CDs are expected to be applied to cell imaging. To determine whether CDs can be used as biological probes, we examined the cytotoxicity of HGC-27 cells after treatment with CDs. As shown in Supplementary Figure S9, after 24 h of incubation, the viability of HGC-27 cells is maintained above 76%. After 48 h of incubation, the viability of HGC-27 cells is maintained above 72%, indicating that the prepared CDs have good biocompatibility and can be used for biological applications. In addition, we investigated the *in vitro* bioimaging of HGC-27 cells containing CDs using confocal techniques (Figures 7B–D) (Liu et al., 2020) Cells incubated with CDs exhibit intense green and red fluorescence under 465–495 nm and 512–552 nm excitation, respectively. Therefore, the prepared CDs with beneficial biocompatibility can be applied in the field of biological cell imaging (Huang et al., 2021).

## 4 Conclusion

In summary, we developed a facile method for synthesizing CDs from agricultural waste. The synthesized CDs possessed the advantages of good stability, low cost, green, and they exhibited a large Stokes shift with an emission wavelength of 480 nm under 365 nm excitation. The CDs can be used as fluorescent nanosensors for the detection of Fe^3+^ ions. The detection limit (63 nM) for Fe^3+^ in water is much lower than WHO recommended value. In addition, we also demonstrated that the CDs have low cytotoxicity and excellent biocompatibility, and the CDs can be applied for living cell imaging. We believe that the synthesis of CDs using agricultural waste conforms to the concept of green chemistry and is a green and environmentally friendly synthesis method, and will pave a new way for a rational use of agricultural waste resources.

## Data Availability

The raw data supporting the conclusion of this article will be made available by the authors, without undue reservation.
