# Supplementary material for: Stalk-derived carbon dots as nanosensors for Fe3+ ions detection and biological cell imaging
Source: Front Bioeng Biotechnol. 2023 Apr 28;11:1187632. doi: 10.3389/fbioe.2023.1187632 (PMC10175696; doi:10.3389/fbioe.2023.1187632)
Supplement: Supplementary file 1 [file Table1.DOCX]

Supplementary Material

Stalk-derived carbon dots as nanosensors for Fe^3+^ ions detection and biological cell imaging

Yongchao Du, Yaxi Li, Yunliang Liu, Naiyun Liu*, Yuanyuan Cheng, Qiuzhong Shi, Xiang Liu*, Zhimin Tao, Yumeng Guo, Jianguo Zhang, Najmeh Askaria and Haitao Li*

*** Correspondence:** Naiyun Liu: liuny@ujs.edu.cn, Xiang Liu: liuxiang0222@126.com, Haitao Li: liht@ujs.edu.cn

**Pre-treatment process of CSP**

First, the carbon material was prepared from CSP. The CSP was soaked with 1% sodium sulfide for 4 hours at 40°C, and then washed several times with ultrapure water until the solution was neutral. An appropriate amount of washed corn stalk was added to a solution containing 20% CH_3_OH and 0.2% HCl. The mixture was then transferred to a Teflon-lined autoclave and kept at 160 °C for 30 min. After cooling to room temperature, the mixture was then centrifuged at 10000 rpm for 3 min and washed with ultrapure water several times. Afterwards, the pretreated powder can be obtained by drying in an oven at 60°C for more than 12 h.


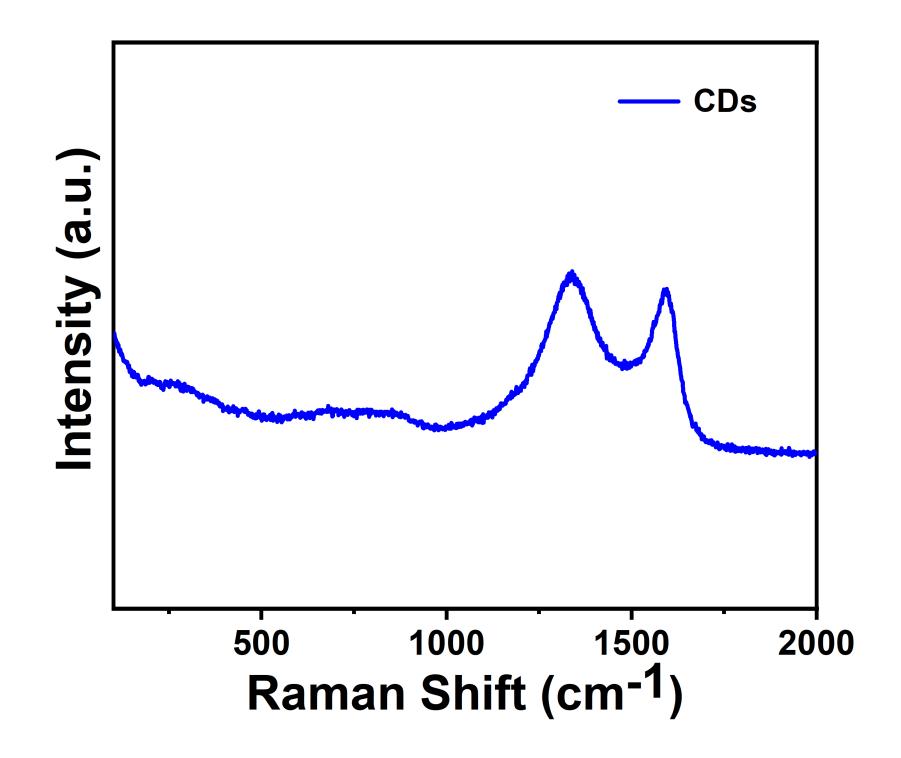


**Supplementary Figure 1.** Raman spectrum of CDs.


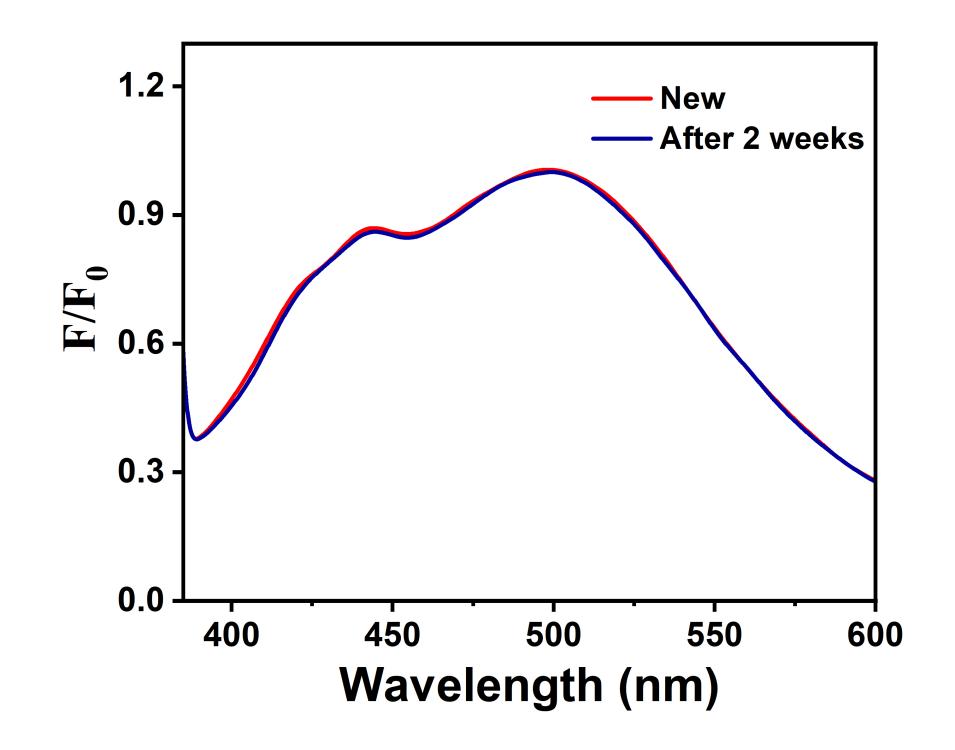


**Supplementary Figure 2.** Comparison of the fluorescence intensity of new prepared CDs and CDs placed for two weeks.


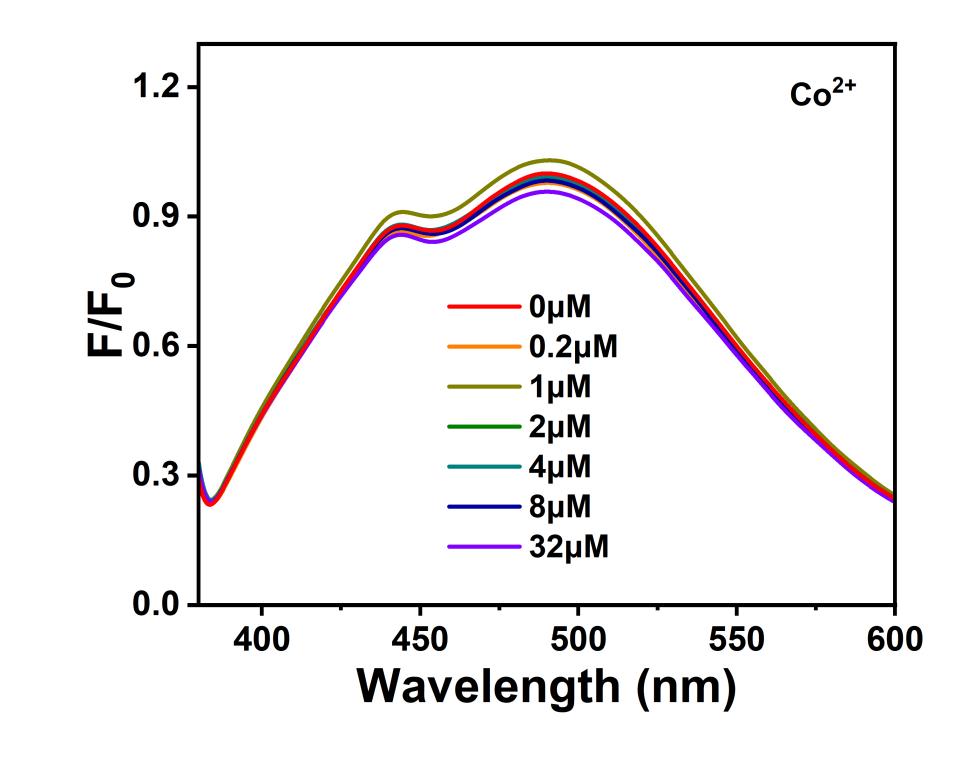


**Supplementary Figure 3.** The relationship between the addition of different concentrations of Co^2+^ ions and fluorescence intensity of CDs solution.


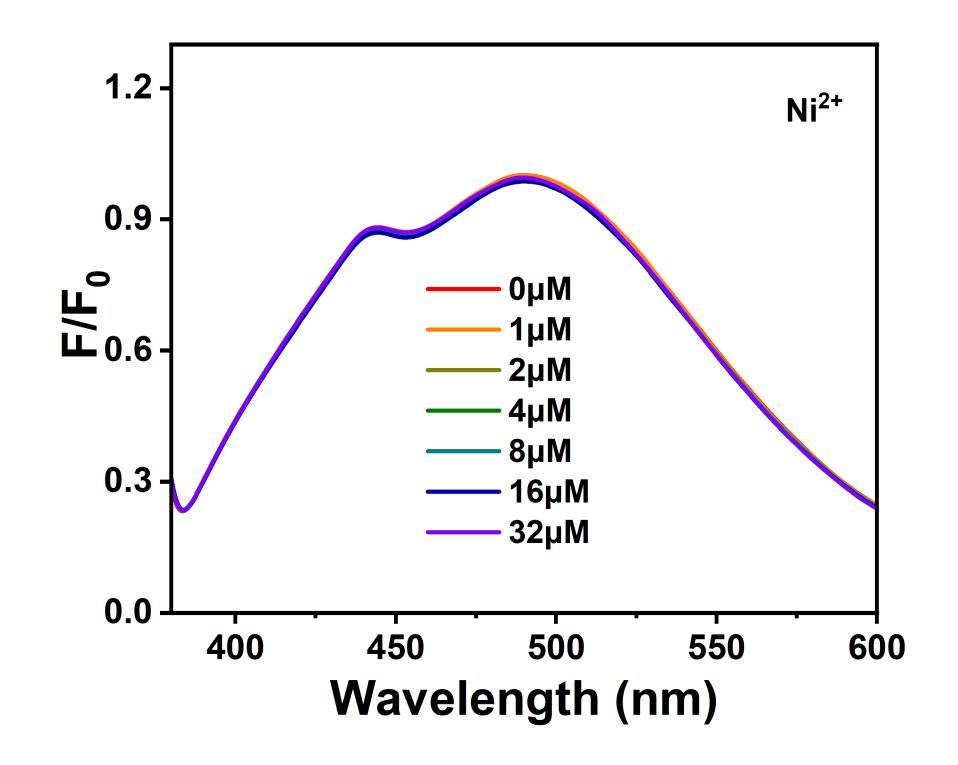


**Supplementary Figure 4.** The relationship between the addition of different concentrations of Ni^2+^ ions and fluorescence intensity of CDs solution.


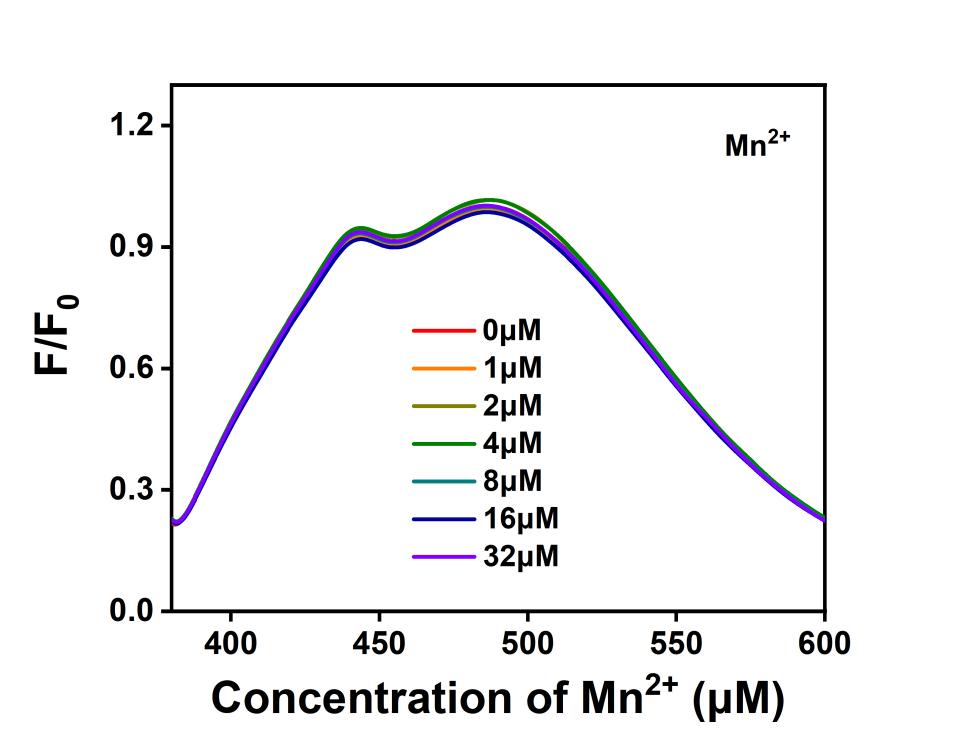


**Supplementary Figure 5.** The relationship between the addition of different concentrations of Mn^2+^ ions and fluorescence intensity of CDs solution.


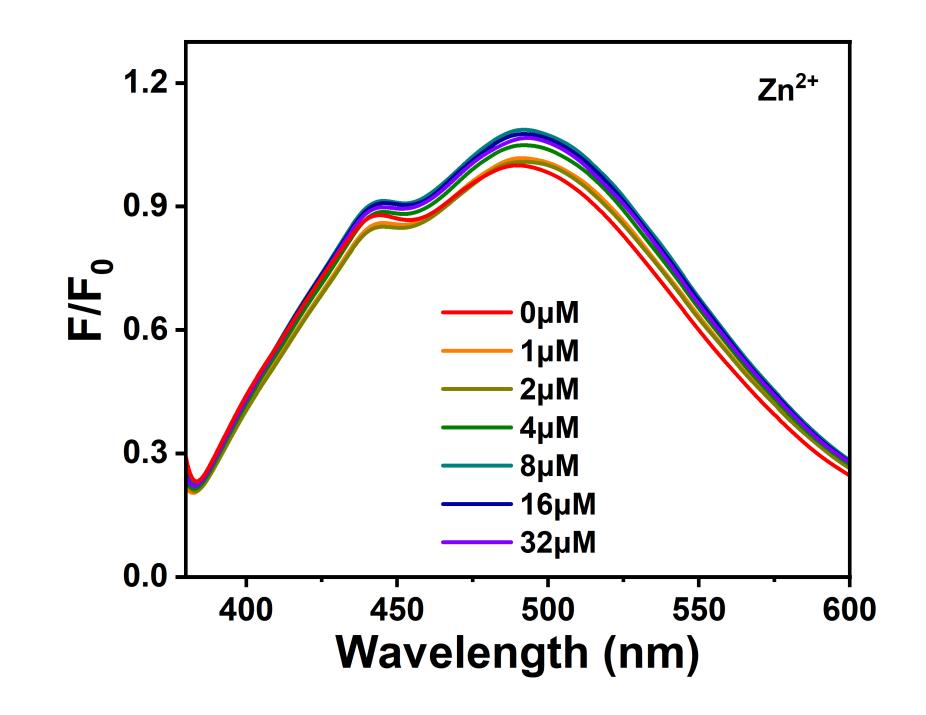


**Supplementary Figure 6.** The relationship between the addition of different concentrations of Zn^2+^ ions and fluorescence intensity of CDs solution.


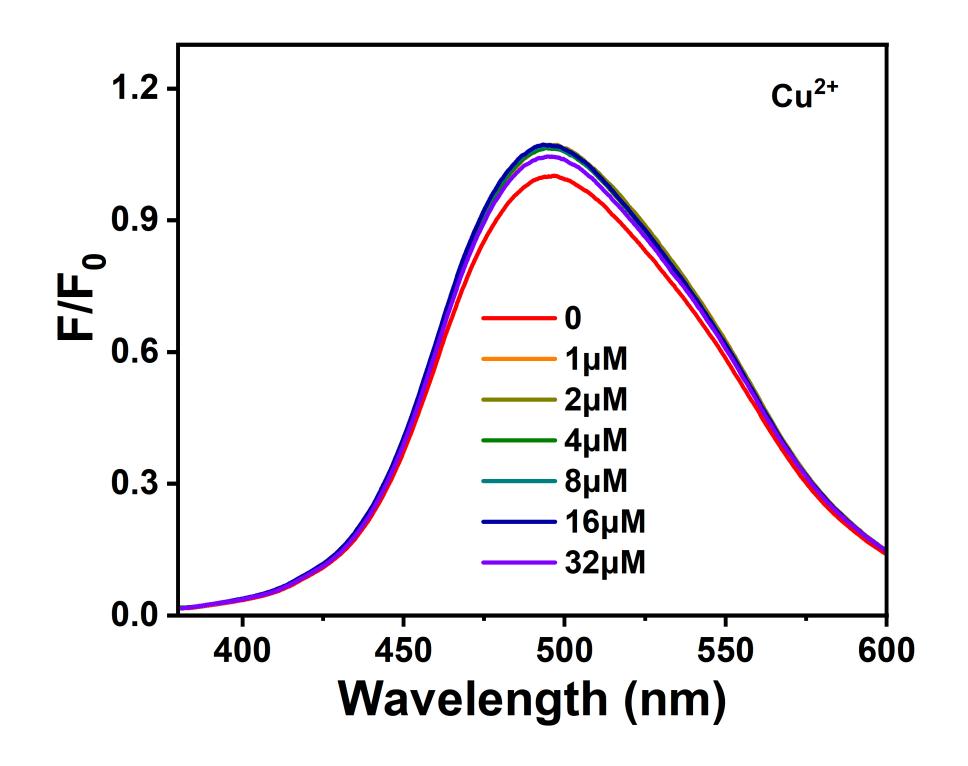


**Supplementary Figure** **7.** The relationship between the addition of different concentrations of Cu^2+^ ions and fluorescence intensity of CDs solution.


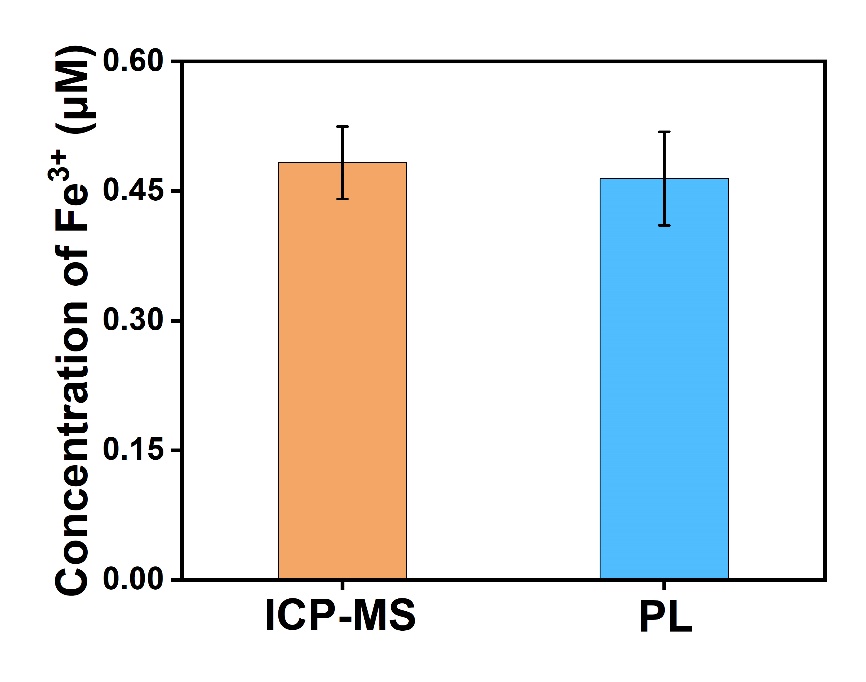
**Supplementary Figure** **8.** The concentration of Fe^3+^ in tap water tested by ICP-MS and fluorescence quenching method.


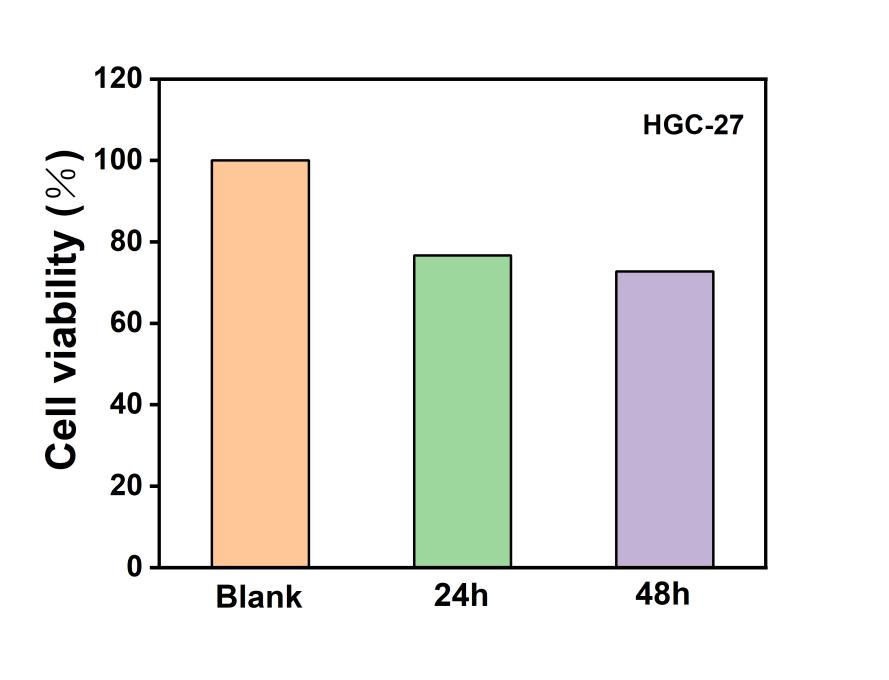


**Supplementary Figure** **9.** Cellular activity after incubation with CDs solution.
